# Effect of urea as a chaotropic agent on self-association of organic molecules in aqueous flow batteries

**DOI:** 10.1039/d5cp03782d

**Published:** 2026-02-12

**Authors:** Mahsa Shahsavan, Cedrik Wiberg, Aapo Poskela, Eduardo Martínez-González, Pekka Peljo

**Affiliations:** a Department of Mechanical and Materials Engineering, Faculty of Technology, University of Turku 20014 Turku Finland pekka.peljo@utu.fi; b Rivus Batteries, Medicinaregatan 8B 41390 Gothenburg Sweden; c Department of Chemistry and Materials Science, Aalto University, Kemistintie 1 02150 Espoo Finland pekka.peljo@aalto.fi

## Abstract

This paper investigates the effect of urea, a widely used denaturing co-solute, on the aggregation of promising candidates for aqueous organic flow batteries, specifically 9,10-anthraquinone-2,7-disulfonic acid (AQDS) and naphthalene diimide derivatives (NDIs). These molecules undergo aggregation through π–π interactions of their aromatic cores. We evaluated how urea influences molecular interactions and electrochemical behavior of these molecules by nuclear magnetic resonance (NMR), cyclic voltammetry (CV), rotating disk electrode (RDE), and flow battery testing. While NMR confirmed that urea effectively disrupts π–π stacking and reduces the concentration-dependent shifts and peak broadening, electrochemical measurements showed that this effect is only partial. These results highlight the difference between molecular-level disruption of aggregation and limited improvements in electrochemical performance.

## Abstract 2

Urea reduced the aggregation of redox active molecules, but did not lead to improved capacity utilization.

## Graphical abstract

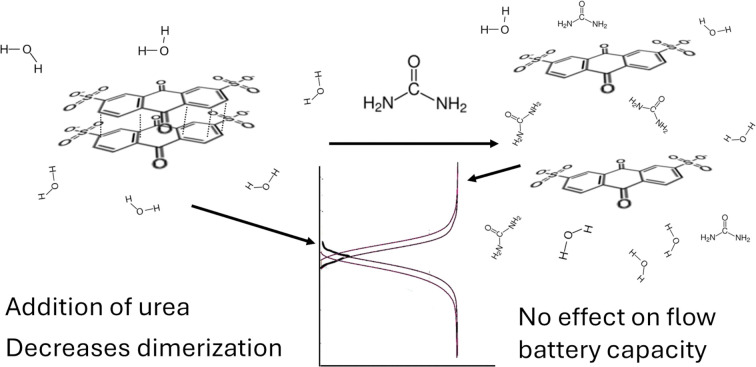

## Introduction

Flow batteries have gained attention as a promising solution for large-scale energy storage due to their scalability, long cycle life, and decoupled energy and power capacities.^1^ Most commercial flow batteries rely on vanadium-based electrolytes; however, concerns over vanadium's cost, toxicity, and limited supply have driven interest toward organic molecules as alternatives.^2^ Among various redox-active organic molecules, quinones have emerged as strong candidates because of their fast redox kinetics, structural tunability, and ability to undergo two-electron redox reactions that increase the energy density of the battery.^3,4^ However, one of the key limitations of quinones or other organic molecules with an aromatic core is their tendency to self-associate in aqueous solutions through π–π stacking interactions.^5^ At high concentrations in aqueous solutions, the well-known negolyte molecule 9,10-anthraquinone-2,7-disulfonic acid (AQDS) aggregates under near-neutral and acidic conditions. This aggregation has been hypothesized to be directly linked to the experimentally observed capacity reduction as AQDS can deliver only about 75% of its theoretical capacity in carbonate buffer.^1,6–9^ Recently, researchers have studied the effects of substituents on the aggregation and accessible capacity. A recent study has examined substituent effects in a series of anthraquinone derivatives and showed that, in the presence of carbonate ions, the number of electrons exchanged per molecule in an electrochemical reaction decreases as a result of AQDS-CO_2_ adduct formation that further limits the charge utilization.^10^ In addition to quinones, another family of organic molecules that has recently been studied for use in flow batteries is the naphthalene diimide (NDI) family. Like quinones, NDIs also exhibit self-association behavior.^6^ However, this phenomenon does not seem to compromise the capacity utilization in all of the NDI flow batteries as compared to AQDS.^11–13^ Regardless, researchers are exploring approaches to enhance capacity utilization of the organic molecules used in aqueous flow batteries. One suggested approach is to minimize their tendency for self-association by modifying the molecular structure.^14^ An alternative strategy explored in this work is the use of additives that can interfere with molecular aggregation. In this context, urea was selected. Due to its polar functional groups, urea readily forms hydrogen bonds with water molecules and acts as a denaturing co-solute. Rather than disrupting the bulk hydrogen-bond network of water, urea primarily operates through local urea–water interactions that alter the dynamics of nearby water molecules.^15^ At sufficiently high concentrations, these local interactions weaken hydrophobic driving forces and shift the aggregation equilibrium toward monomeric species, while the overall hydrogen-bond structure of bulk water remains largely preserved.^16^ For example, urea can reduce the aggregation and increase the solubility of Azure B molecules in aqueous solutions, affect the solvation energy of ions and increase the solubility of flow battery electrolytes.^17–19^ Additionally, in 2020, it was reported that urea can stabilize concentrated 4-amino-1,1′-azobenzene-3,4′-disulfonic acid monosodium salt (AADA) electrolytes through anisotropic hydrogen bonding, supported by Raman spectroscopy and DFT calculations.^20^ While the effect of urea has been studied on some molecules, its effect on the aggregation of AQDS and NDIs in the context of flow batteries remains unreported. Therefore, in this study, we investigated the effect of urea on the self-association and electrochemical behavior of AQDS and two NDI derivatives as illustrated in Table 1 using nuclear magnetic resonance (NMR), cyclic voltammetry (CV), rotating disk electrode (RDE), and flow battery testing. Urea was found to disrupt π–π stacking at the molecular level, but only partially suppressed the aggregation in electrochemical measurements and resulted in minor improvements in flow battery performance. Moreover, urea is not electrochemically active on carbon electrodes at near-neutral pH used in this study.^21^

**Table 1:** Molecular structures and redox reactions of the redox-active species investigated in this work, where O is the oxidized form, R_1_ is the singly reduced form, R_2_ is the result of the second reduction and R is the reduced AQDS where the –C=O groups of the quinone are converted to the hydroquinone form –C–OH

D-NDI^6^	GABA-NDI^12^	AQDS^1^
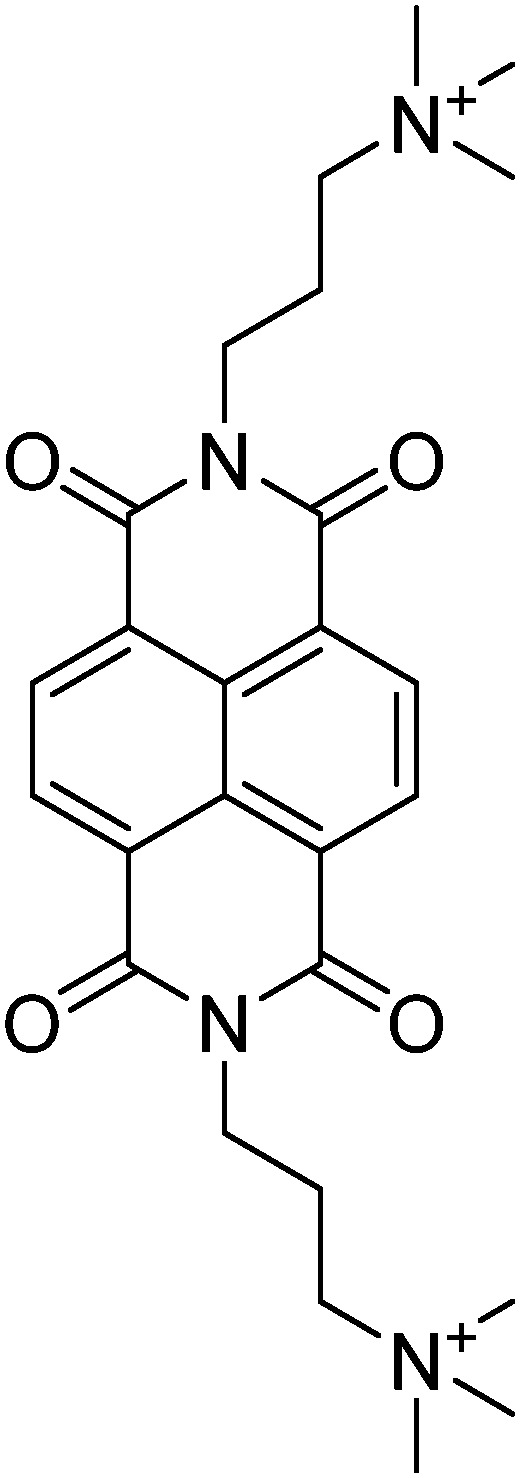	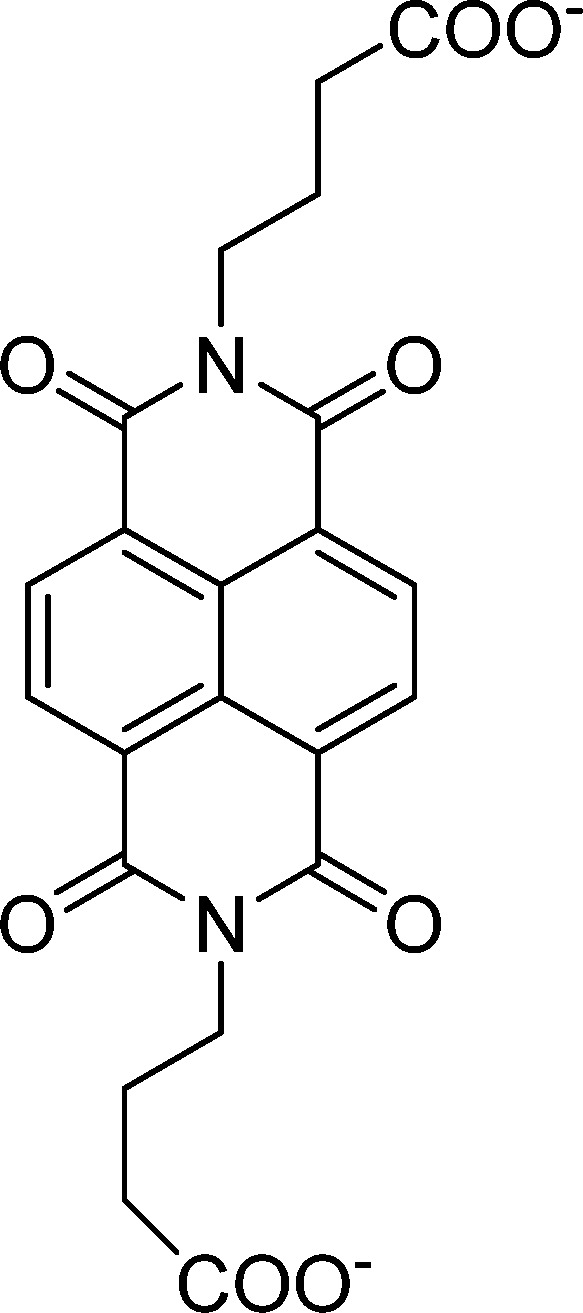	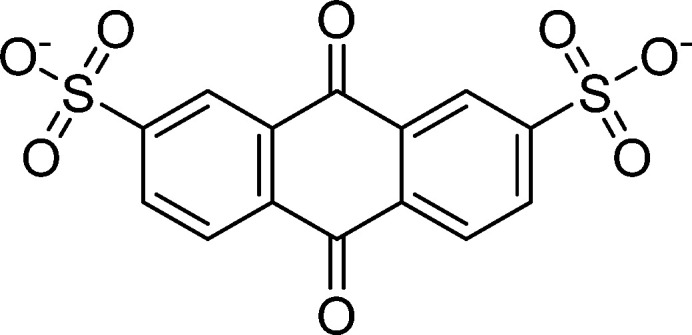
O^2+^ + e^−^ ↔ R_1_^+^	O^2−^ + e^−^ ↔ R_1_^3−^	O^2−^ + 2e^−^ + 2H^+^ ↔ R^2−^
R_1_^+^ + e^−^ ↔ R_2_	R_1_^3−^ + e^−^ ↔ R_2_^4−^	

## Results and discussion

### Nuclear magnetic resonance (NMR) spectroscopy

**Fig. 1:**
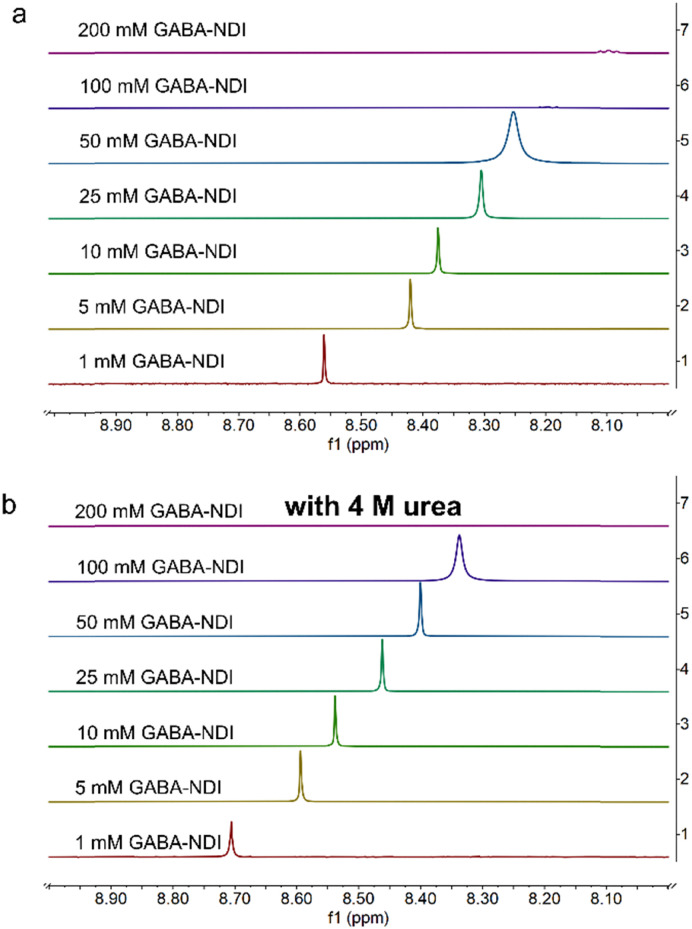
^1^H-NMR spectra of the aromatic peak for a concentration series of GABA-NDI in 1 M NH_4_Cl in 10% D_2_O (a) without urea and (b) with the addition of 4 M urea.

**Fig. 2:**
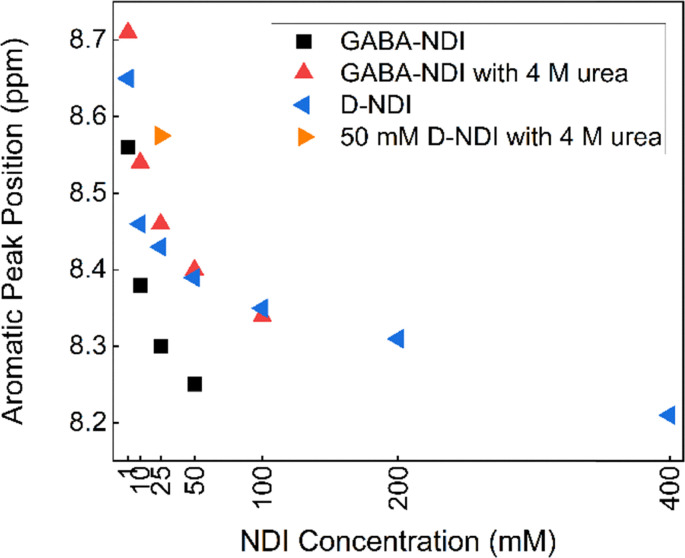
Chemical shift positions of the aromatic peak of NDIs as a function of concentration.

In this work, the self-association was evaluated by monitoring changes in the chemical shift and peak broadening of the aromatic proton signals in the case of NDI derivatives, and changes in the chemical shifts in the case of AQDS. These changes were analyzed as a function of both the concentration of the studied molecule and the concentration of urea in the solution. The NMR spectra of GABA-NDI at varying concentrations in Fig. 1a (full spectra in Fig. S3) revealed clear concentration-dependent chemical shift changes in the aromatic region that is consistent with aggregation at higher concentrations meaning that increased aggregation leads to greater shielding and consequently an upfield shift of the aromatic peaks.^6^ Peak broadening was also observed with increasing concentrations above 100 mM, and the aromatic peak became severely broadened and eventually merged into the baseline due to the presence of large aggregates. Upon the addition of 4 M urea, the concentration-dependent shifts were diminished (Fig. 1b and Fig. S4, S5). The shift of the aromatic peak as a function of concentration without and with 4 M urea shown in Fig. 2 clearly shows this behavior.

**Fig. 3:**
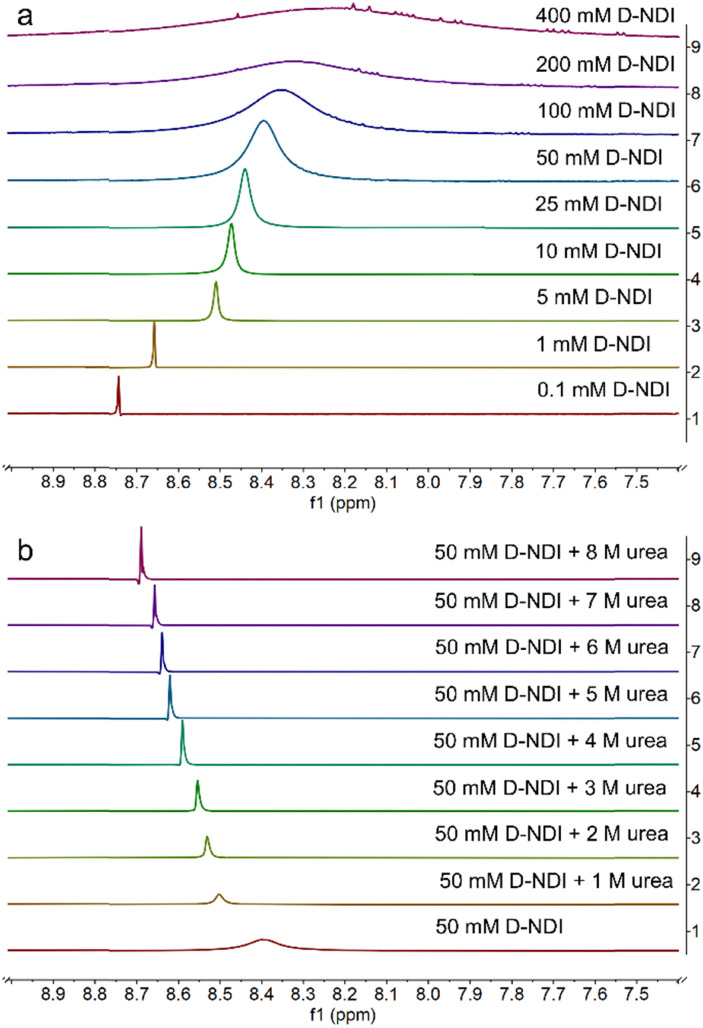
^1^H-NMR spectra of the aromatic peak (a) for a concentration series of D-NDI and (b) 50 mM D-NDI with a concentration series of urea in 500 mM phosphate buffer of pH 7 in 10% D_2_O.

**Fig. 4:**
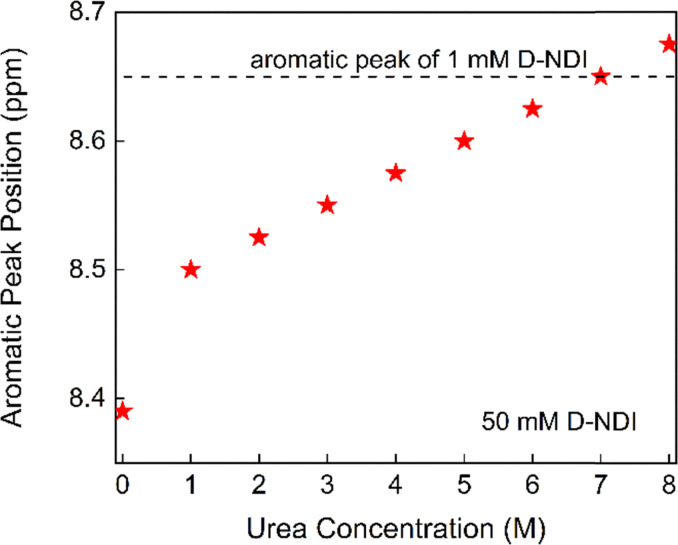
Chemical shift positions of the aromatic peak of 50 mM D-NDI as a function of different urea concentrations.

For D-NDI in Fig. 3a, the same trend observed for GABA-NDI is present. The aromatic peak of GABA-NDI disappeared already at 100 mM, where the aromatic peak merges into the baseline, whereas in D-NDI this occurs around 400 mM. However, the peak broadening described by full width at half maximum (FWHM) in Fig. S6 shows order of magnitude higher broadening for D-NDI. Next, the effect of different concentrations of urea in 50 mM D-NDI solutions is shown in Fig. 3b. The increasing amount of urea sharpened the peaks and caused a gradual downfield shift close to the diluted solutions of the same molecule (Fig. 4). Overall, the NMR data confirm that both GABA-NDI and D-NDI self-associate in aqueous media through π–π stacking, and that urea can effectively disrupt these aggregates and bring the spectra closer to the monomeric states of the molecules.

**Fig. 5:**
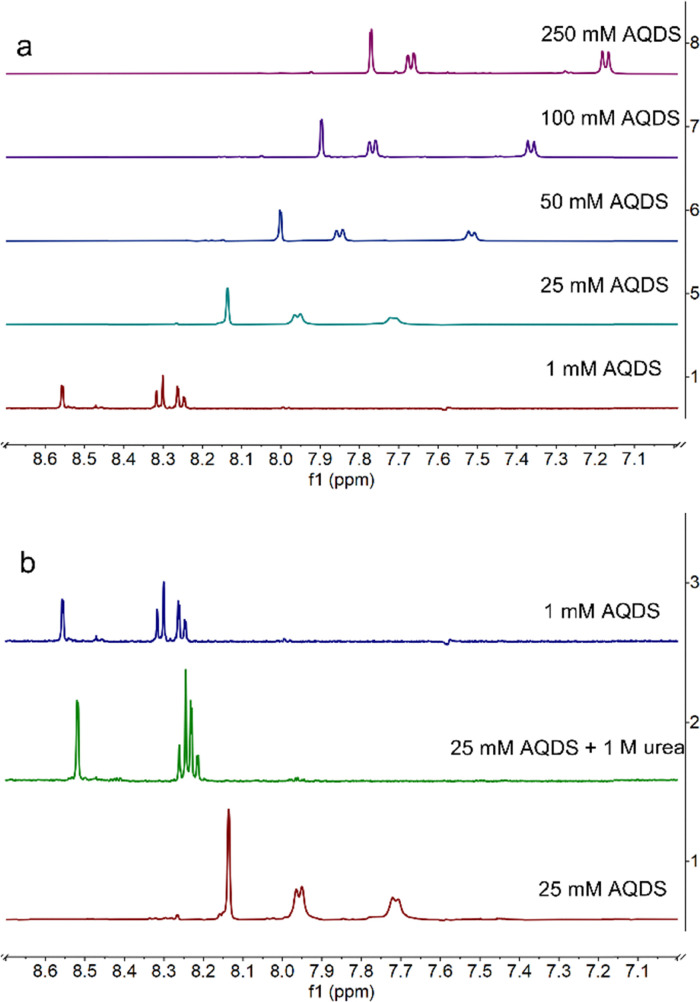
^1^H-NMR spectra on a (a) concentration series of AQDS and (b) 25 mM AQDS with/without urea in comparison to 1 mM AQDS in 1 M sodium carbonate buffer pH 9.5 in 10% D_2_O.

**Fig. 6:**
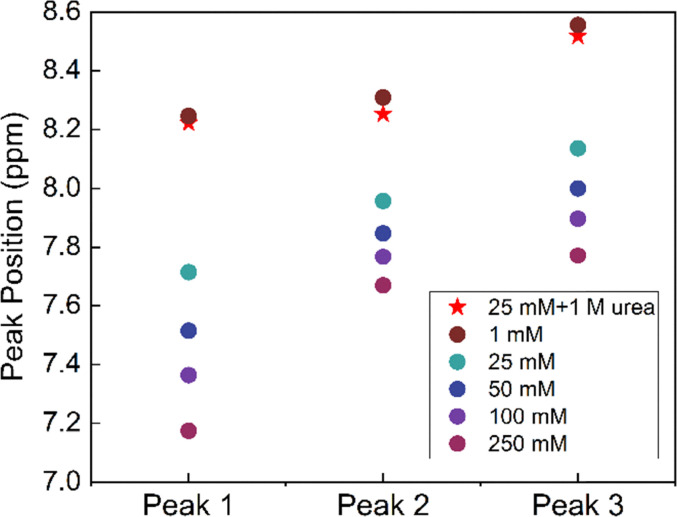
Chemical shift positions of AQDS peaks as a function of concentration, with/without 1 M urea.

Next, the evolution of the NMR spectrum of AQDS was studied in the range of 1–250 mM. As shown in Fig. 5a, increasing the concentration of AQDS resulted in an upfield shift of the peaks which is a result of the enhanced π–π stacking interactions between AQDS molecules at higher concentrations.^8^ NMR spectra were recorded on 25 mM AQDS in the presence and absence of 1 M urea (Fig. 5b). In the absence of urea, the peaks were shifted upfield relative to the spectra for 1 mM AQDS; however, upon the addition of 1 M urea, the peaks shifted downfield, became noticeably sharper and at the same time approached the chemical shifts observed for 1 mM AQDS. The concentration-dependent chemical shift data of the peaks are shown in Fig. 6. All three peaks exhibited a consistent downfield shift with increasing concentration, while the presence of 1 M urea shifted the peaks toward higher ppm values.

According to these NMR results, urea as an additive can reduce the driving force for π–π stacking around aromatic cores through local urea–water and urea–solute interactions that modify the solvation environment, as explained in the literature.^15,22,23^ Explicit-solvent molecular dynamics simulations have demonstrated that urea suppresses aggregation by preferentially stabilizing exposed solute states through direct urea–solute interactions rather than by disrupting bulk water.^15,24^ In addition, UV-Vis studies on hydrophobic dyes have shown urea-induced de-aggregation and solubilization consistent with preferential interaction models.^22^ The effect of increasing urea concentration has been examined in the literature at the molecular level using spectroscopic and thermodynamic approaches. These studies show that, even at high urea concentrations, the bulk hydrogen-bond network of water remains largely intact, while urea exerts its influence through local interactions in its hydration environment. Quantitative analyses indicate that only a small, well-defined fraction of water molecules is directly affected by urea through hydrogen bonding (approximately one to two water molecules per urea), and this hydration number remains nearly independent of urea concentration. In parallel, the increasing urea concentration enhances local modifications of hydration dynamics, such as shortened hydrogen-bond lifetimes and an increased population of non-hydrogen-bonded water molecules in the immediate vicinity of urea. Thus, increasing the urea concentration does not correspond to a progressive increase in bulk chaotropic extent, but rather to a greater statistical contribution of these local urea–water and urea–solute interactions. These local effects weaken hydrophobic interactions and shift aggregation equilibria toward monomeric species, in agreement with our experimental observations.^15,22,25^ Based on this, we believe that the same approach could be utilized to regulate the aggregation of other organic molecules such as viologens, phenazines, *etc.*

### Cyclic voltammetry

**Fig. 7:**
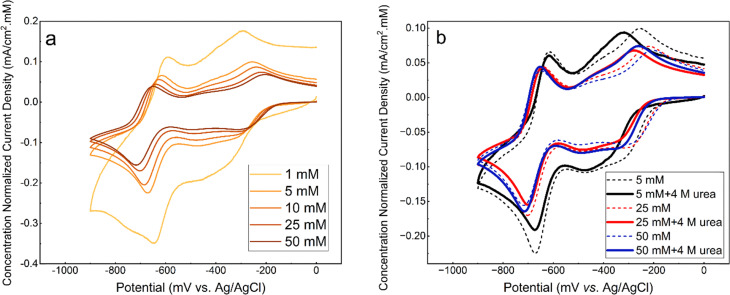
Concentration-normalized CVs of (a) GABA-NDI and (b) GABA-NDI with and without 4 M urea (viscosity corrected) at a scan rate of 100 mV s^−1^ in 1 M NH_4_Cl.

In cyclic voltammetry experiments, peak current and reduction potentials were analyzed to evaluate the impact of urea on the electrochemical behavior of the studied molecules. In order to account for the viscosity-dependent decrease in peak current caused by the addition of urea, all CVs recorded with urea were normalized using a correction factor. The peak current for the reversible process is given by the Randless–Ševčík equation:1
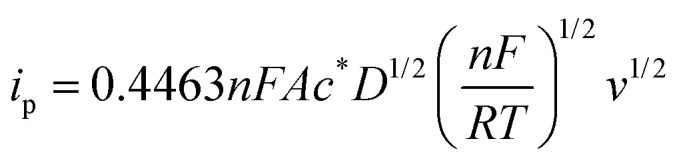
where *n* is the number of electrons, *F* is the Faraday constant, *A* is the area of the electrode, c* is the bulk concentration of the redox species, *D* is the diffusion coefficient, *v* is the scan rate, *R* is a gas constant and *T* is the temperature. As the diffusion coefficient and viscosity are related to each other *via* the Stokes–Einstein relationship (*ηD* = constant), the correction factor becomes the square root of the ratio of the viscosity of the urea-containing solution to that of water. This correction ensures that changes in the peak current is attributable to molecular interactions rather than changes in electrolyte viscosity. Correction factors were calculated using literature-reported viscosity values for water and urea mixtures at 25 °C.^26^ For a solution containing 4 M urea, the corresponding correction factor is 1.10.2
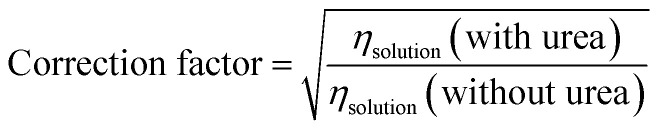
CVs of GABA-NDI in Fig. 7a with two redox reactions show the dependency of the voltammograms on the concentrations. The shape of the voltammogram has been discussed in other publications.^12^ As the GABA-NDI concentration increases, the first reduction shifts towards more positive potentials and the second reduction towards more negative potentials and the peak separations become wider.^6^ This indicates that increased π–π stacking hinders the electron transfer kinetics. The addition of 4 M urea to GABA-NDI solutions in Fig. 7b (Fig. S7) results in a slight decrease in the peak separation. The first reduction shifts towards more negative values upon the addition of urea, and the second reduction is almost unaffected. This behavior can prove that urea can disrupt π–π stacking of the molecules to some extent. However, it does not fully prevent stacking, as the concentration normalized CVs do not overlap, despite the NMR data suggesting otherwise.

**Fig. 8:**
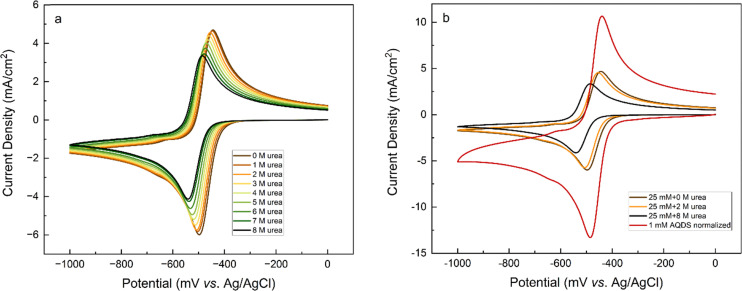
(a) Viscosity corrected cyclic voltammograms of 25 mM AQDS in 1 M sodium carbonate buffer pH 9.5 at a scan rate of 100 mV s^−1^, (b) comparison of CVs for 25 mM AQDS with and without 2 M urea along with normalized CV of 1 mM AQDS (multiplied by 25).

**Fig. 9:**
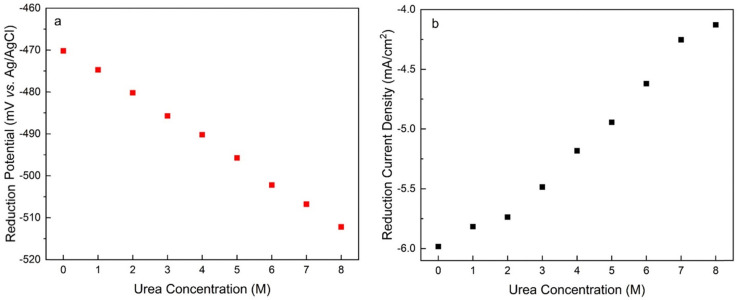
(a) Reduction potential and (b) reduction peak current density of 25 mM AQDS plotted as a function of urea concentration (viscosity corrected).

CVs of 25 mM AQDS solutions with different concentrations of urea are shown in Fig. 8a (Fig. S8). In the absence of urea, AQDS displays a redox reaction with a peak separation of *ca.* 55 mV. For AQDS, the number of electrons should be 2, so the peak separation is larger than the expected *ca.* 30 mV, indicating that the reaction can be described as quasi-reversible. Upon the addition of urea, the shape of the voltammograms remains unchanged, but both the reduction and oxidation peaks gradually shift towards more negative potentials with increasing urea concentration with peak separation varying sporadically between 53 and 57 mV (Fig. S9). Fig. 9a shows that the decrease in the redox potential with the addition of urea is almost linear and indicates that the oxidized form of the AQDS becomes harder to reduce.^27^Fig. 9b shows that the peak current decreases more than expected based on only increased viscosity. This might also be affected by temperature and the presence of the electrolyte.

Comparison of the normalized CVs of 1 mM AQDS and 25 mM AQDS in the presence of urea in Fig. 8b shows that urea only partially disrupts stacking. Like the case of GABA-NDI, if stacking was fully prevented, the peak currents for 25 mM AQDS with urea should match that of the 1 mM AQDS, but it is *ca.* half of the expected value. Finally, the effect of urea on the cyclic voltammograms of the ferro/ferricyanide redox couple was also evaluated, as this system is used in the subsequent flow battery experiments (Fig. S14). The cyclic voltammograms of the ferro/ferricyanide redox couple exhibit a peak-to-peak separation of approximately 83 mV at 100 mV s^−1^ in the absence of urea indicating quasi-reversible electron-transfer kinetics. Upon increasing the urea concentration, both anodic and cathodic peak currents decrease accompanied by an increase in peak-to-peak separation that reaches 108 mV in the presence of 8 M urea. These results indicate that urea hinders charge-transfer kinetics. The redox potential of ferro/ferricyanide does not shift, indicating that for AQDS the addition of urea affects the dimerization, making the oxidized form more stable and shifting the redox potentials towards negative values.

### Rotating disk electrode

**Fig. 10:**
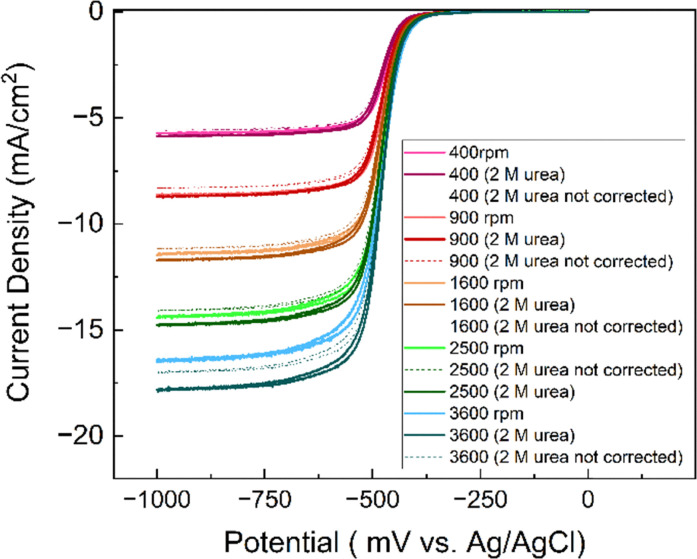
RDE voltammograms of 25 mM AQDS at varying rotation speeds with/without urea in 1 M sodium carbonate buffer pH 9.5 at a scan rate of 1 mV s^−1^ corrected by viscosity.

**Fig. 11:**
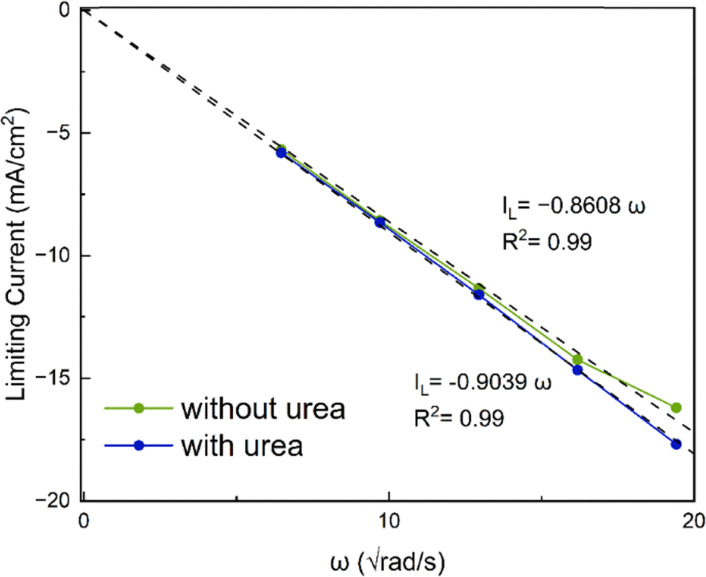
Levich analysis on AQDS.

In rotating disk electrode measurements, the limiting currents were used for analysis. However, addition of urea to the solution increases its viscosity and decreases the diffusion coefficient of the molecules and the limiting current. The limiting current (*i*_lim_) in RDE experiments is given by the Levich equation:

3 *i*_lim_ = 0.62*nFAD*^2/3^*ω*^1/2^*ν*^−1/6^c*

where *n* is the number of electrons, *F* is the Faraday constant, *A* is the area of the electrode, *D* is the diffusion coefficient, *ω* is the rotating rate, *ν* is the kinematic viscosity and c* is the bulk concentration of the redox active species. Upon aggregation, the concentration of the active species reduces but the number of electrons should increase correspondingly (the dimer should be reduced by 4 electrons). The effective concentration of redox species can be given as nc* = *fnc*_0_ where the *c*_0_ is the initial concentration of AQDS, *n* = 2 and *f* is the fraction of accessible capacity, with values between 0 and 1. Additionally, the diffusion coefficient of aggregates is decreased in comparison to monomers. To be able to compare RDE data, the measured currents were normalized to account for the viscosity differences in the measurements with urea with a correction factor.4
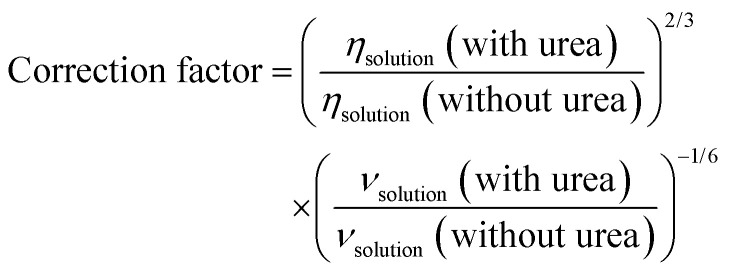
For a solution containing 2 M urea, the corresponding correction factor is 1.047. According to Fig. 10, at all rotation rates, the limiting current is slightly higher in the presence of urea. Comparison of RDEs for the normalized 1 mM and 25 mM AQDS in Fig. S10, shows lower currents for 25 mM AQDS indicating that the accessible monomer concentration for the reaction is less.^7^ When urea is introduced, this loss of limiting current is partially recovered. This suggests that urea enhances the accessible monomer concentration by disrupting aggregation. Without urea, AQDS molecules form aggregates, reducing the effective concentration of an electroactive monomer in solution. With urea, aggregation is suppressed, leaving more AQDS monomers available in the solution to react. This results in higher measured current densities.^28^ Using the Levich equation and the accessible concentration for 25 mM AQDS reported by Wiberg *et al.* that is 19.1 mM,^7^ we got a diffusion coefficient *D* = 2.11 × 10^−6^ cm^2^ s^−1^ for 25 mM AQDS solution, with *f* = 0.76. If we use the diffusion coefficient corrected by viscosity (*D* = 1.95 × 10^−6^ cm^2^ s^−1^ in the presence of urea), the measured limiting currents correspond to an accessible concentration of 21.6 mM for 25 mM AQDS solution meaning that around 86% of AQDS molecules are in electrochemically accessible form. Thus, the RDE results are consistent with the NMR and CV results and confirm that urea can reduce aggregation of AQDS in solution to some extent, leading to a higher effective concentration of redox-active species available for the redox reaction (Fig. 11).

### Flow battery measurements

**Fig. 12:**
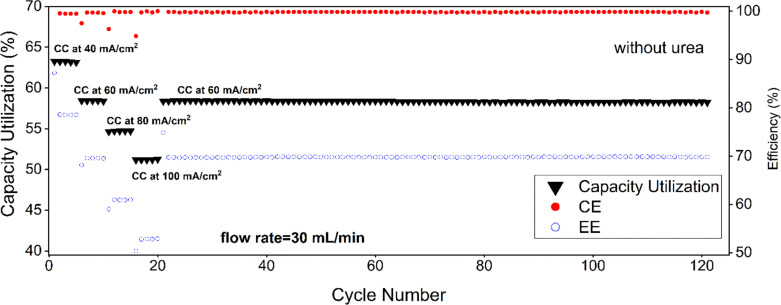
Flow battery performance of 20 mL of 25 mM AQDS with 50 mL of 25 mM sodium ferrocyanide (Na_4_[Fe(CN)_6_]) in 1 M sodium carbonate buffer pH 9.5 without urea.

**Fig. 13:**
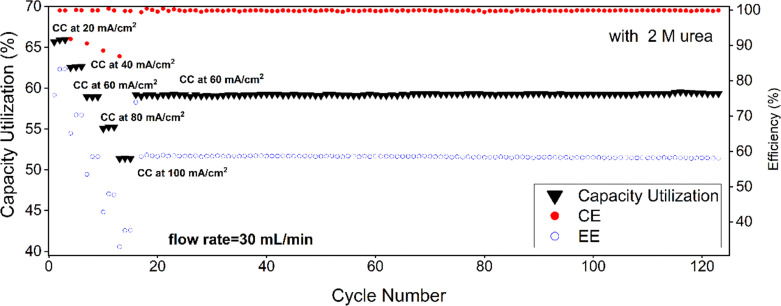
Flow battery performance of 20 mL of 25 mM AQDS with 50 mL of 25 mM Na_4_[Fe(CN)_6_] in 1 M sodium carbonate buffer pH 9.5 with 2 M urea.

Flow battery measurements (Fig. 12 and 13) can finally confirm the effect of urea on the electrochemistry of AQDS in a battery. Without urea, capacity utilization is 63% at 40 mA cm^−2^ and drops down to 51% at 100 mA cm^−2^ (Fig. 12 and 13). Coulombic efficiency (CE) remains close to 100% across all current densities, indicating negligible side reactions and the energy efficiency (EE) is 70% for long cycling at 60 mA cm^−2^. The energy efficiencies decrease from 79, 70, 61 and 53% without urea to 70, 59, 48, and 38% with urea when increasing the current density by 20 mA cm^−2^ steps from 40 to 100 mA cm^−2^. This decrease is linear with slopes of −0.43 and −0.57% per 1 mA cm^−2^ without and with 2 M urea, respectively. The addition of urea was found to have a minimal effect on capacity utilization across all current densities. At 100 mA cm^−2^, capacity utilization with urea is 51.4%, compared to 51% without urea although RDE measurements indicate that a capacity close to 86% should be achieved. Coulombic efficiency (CE) remains close to 100%, confirming that urea does not introduce parasitic reactions. However, the energy efficiency (EE) drops down to 58% for long cycling at 60 mA cm^−2^. The cell high frequency impedance was measured as 0.31 Ω and 0.29 Ω after cell assembly without and with urea, respectively, and the values decreased to 0.24 Ω and 0.26 Ω after the cycling (Fig. S13). These small differences in cell resistance cannot explain such a drop in energy efficiency, and instead this difference results from the decreased kinetics of the ferro/ferricyanide couple in the presence of urea, as discussed in the next section.

The average cell voltage is *ca.* 720 mV both with and without urea. By considering the resistance of the cell, in the absence of urea, *ca.* 60% of the losses is estimated to result from Ohmic resistance (considering *R* = 0.24 Ω, cycling at 60 mA cm^−2^*i.e.* at 300 mA results in average charge voltage of 790 mV and discharge voltage of 650 mV, giving energy efficiency of 82% when the Coulombic efficiency is 100%) while 40% would result from activation losses to reach 70% energy efficiency (the total loss is 30%, 18 percentage units from *iR* and the rest from activation). With urea, the *iR* contribution changes to 48% (the total loss is 42%, 20 percentage units from *iR* considering *R* = 0.26 Ω and the rest from activation). The presence of 2 M urea doubles the activation losses.

### Effects of urea on flow battery performance

Our results indicate that addition of urea decreases the aggregation of organic molecules but does not affect the capacity utilization in a flow battery. However, the redox potentials of NDIs and AQDS decrease while the kinetics of the electron transfer can be positively affected. Interestingly, kinetics of the ferri/ferrocyanide couple decrease upon the addition of urea while the redox potential is not affected. Moreover, the addition of urea slightly increases the measured resistance of the cell, explained by the increased viscosity affecting also ionic conductivity. The energy efficiency of the AQDS battery decreased from 70% to 58% at 60 mA cm^−2^. The reason for this drop can be explained by the decreased kinetics of the ferro/ferricyanide couple as well as a slight increase in the resistance (Fig. S14). Additionally, the increased viscosity due to addition of urea results in decreased convective mass transfer.^29^ These effects arise from altered solvation of the molecules in the presence of urea and increased electrolyte viscosity leading to reduced convection and increased concentration polarization that directly affect the voltage efficiency and the energy efficiency of the system, but are difficult to quantify without simulations. Overall, the addition of urea to the battery electrolyte has little impact on the capacity utilization. This suggests that aggregation alone cannot fully explain the limited utilization of AQDS. Carney *et al.* hypothesized that adduct formation between AQDS and CO_2_ significantly affects the charge accessibility.^8^ Pasadakis-Kavounis *et al.* demonstrated that capacity loss in AQDS systems arises from the CO_2_-induced increase of the oxidation potential: CO_2_–adduct formation with AQDS causes the re-oxidation process of AQDS to split in two separate 1-electron steps. As the second electron step at a significantly more positive potential lies outside of the potential range accessed during battery operation, reduced capacity is accessed in typical battery experiments.^10^ Therefore, even though urea can suppress aggregation, the dominant capacity-limiting process in our carbonate buffer appears to be CO_2_–adduct formation, which is unaffected by urea. This explains the lack of significant improvement in charge utilization despite reduced aggregation. Thus, both aggregation and chemical speciation likely contribute to the observed performance losses. For NDI derivatives, NMR results confirm that urea can disrupt their self-association. However, since NDIs already display high capacity utilization in the flow batteries reported in the literature, self-association does not appear to limit their performance in a flow battery as much as it may seem it does in CV or the RDE.^12,13,30^ This can be either because the aggregates are electrochemically active or the dissociation of the aggregates is sufficiently fast so that monomers are constantly generated to replace those that have reacted in the battery. The recent DFT calculations indicate that the dimers are electrochemically active, as the computationally evaluated redox potentials for GABA-NDI and ASP-NDI match the experimental measurements only if dimerization is considered.^13^ Therefore, further flow battery testing with urea was not pursued for NDIs. Urea clearly alters the solvation of molecules in electrolytes. While this altered solvation can stabilize monomeric species and mitigate self-association, it can also increase charge-transfer overpotentials and mass-transport resistance through changes in reorganization energy, ion mobility, and electrolyte viscosity. In the following steps, additives that contribute both to stabilization of the monomers and lowering the viscosity should be explored. Finding such candidates can be challenging, as typically additives increase the viscosity of aqueous solutions, but for example co-solvents with lower viscosity are one option. Also, as viscosities decrease with increasing temperature, suitability of urea as an additive at higher temperatures should be investigated.

## Conclusions

This study shows that urea is effective in disrupting π–π stacking at the molecular level, as evidenced by NMR spectra, where concentration-dependent peak shifts and broadening are reversed upon urea addition. We expect that this finding is also applicable to a wider group of organic molecules such as viologens, phenazines, *etc.* However, this suppression of aggregation is less complete than suggested by NMR, as shown by the non-overlapping normalized CV and RDE results. This discrepancy is due to the fact that NMR probes the local chemical environment and is highly sensitive to aggregation, whereas electrochemical techniques show the collective response of all species in solution under dynamic conditions. In these measurements, the observed current reflects not only the balance between monomers and aggregates but also transport phenomena, electron-transfer kinetics, and solution viscosity. Although urea reduces aggregation, a residual fraction of electrochemically inactive aggregates is still in the solution. Furthermore, the increased viscosity of urea-containing electrolytes offsets some of the benefits of reduced aggregation by lowering diffusion coefficients and affecting fluid flow in porous media. Flow battery experiments further confirmed that urea only slightly improves capacity utilization. Importantly, while CE remained unaffected, EE decreased. Thus, while NMR reveals clear molecular-level improvements, these translate into negligible gains in electrochemical performance at the battery level due to the existence of other limiting factors. The capacity of AQDS in carbonate buffer seems to be limited by the formation of the CO_2_ adduct with the reduced AQDS, and as the battery capacity did not improve, we can conclude that urea did not affect this process.

## Author contributions

M. S.: conceptualization, formal analysis, investigation, methodology, validation, visualization, writing – original draft, and writing – review and editing. C. W.: conceptualization, formal analysis, investigation, and methodology. A.P.: visualization, writing – original draft, and writing – review. E. M.-G.: formal analysis, investigation, and writing – review. P. P: conceptualization, formal analysis, funding acquisition, project administration, resources, supervision, writing – original draft, and writing – review.

## Conflicts of interest

The authors have nothing to declare.

## Supplementary Material

CP-028-D5CP03782D-s001

## Data Availability

The data supporting this article have been included as part of the supplementary information (SI). Supplementary information containing description of materials and characterization methods, voltammetry data, and flow battery data is available. See DOI: https://doi.org/10.1039/d5cp03782d.
