# Supplementary material for: Effect of urea as a chaotropic agent on self-association of organic molecules in aqueous flow batteries
Source: Phys Chem Chem Phys. 2026 Feb 12;28(11):6908–16. doi: 10.1039/d5cp03782d (PMC12947893; doi:10.1039/d5cp03782d)
Supplement: CP-028-D5CP03782D-s001 [file CP-028-D5CP03782D-s001.pdf]

This journal is © the Owner Societies 2026

Effect of Urea on Self-Association of Organic Molecules in Flow Batteries

Supplementary information

Mahsa Shahsavan, [a] Cedrik Wiberg, [b] Aapo Poskela, [a] Eduardo Martínez-González, [c] and Pekka Peljo*[a,c]

[a] Department of Mechanical and Materials Engineering, Faculty of Technology, University of Turku, 20014 Turku, Finland

[b] Rivus Batteries, Medicinaregatan 8B, 41390 Gothenburg, Sweden.

[c] Department of Chemistry and Materials Science, Aalto University, Espoo, 00076 AALTO, Finland

* *Corresponding author:* pekka.peljo@utu.fi; pekka.peljo@aalto.fi

Materials:

9,10-Anthraquinone-2,7-disulfonic acid (AQDS) was purchased from BossChemicals with the purity of 98%. Urea, phosphoric acid 85% and trisodium phosphate 96% was purchased from Sigma-Aldrich. Naphthalene diimide derivatives (quaternary amine-functionalized (D-NDI) and gamma aminobutyric acid (GABA-NDI)) were synthesized following previously reported procedures in references (1,2). Stock solutions of AQDS (250 mM), D-NDI (400 mM) and GABA-NDI (200 mM) were prepared in deionized water and diluted to the desired concentrations. Urea was added to the solutions in concentrations from 0 M to 8 M. Deionized water was used to prepare all solution. Purity of AQDS is confirmed with NMR (Figure S1 and Figure S2) (3).

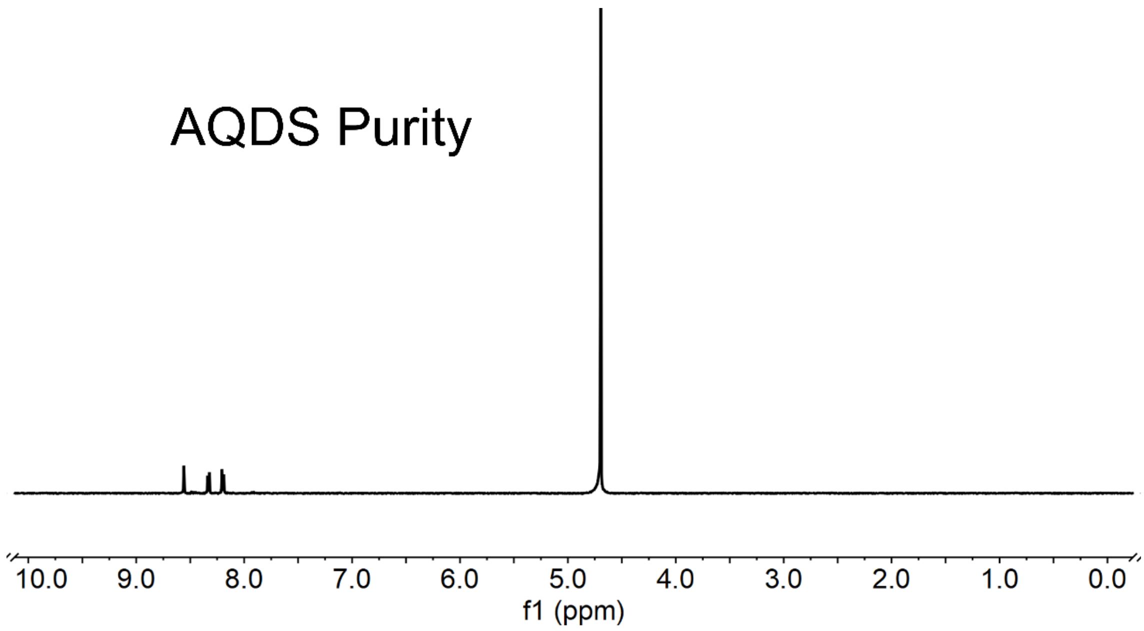

Figure S1- Full ^{1}H NMR spectrum on the purchased AQDS in 10% D_2O.

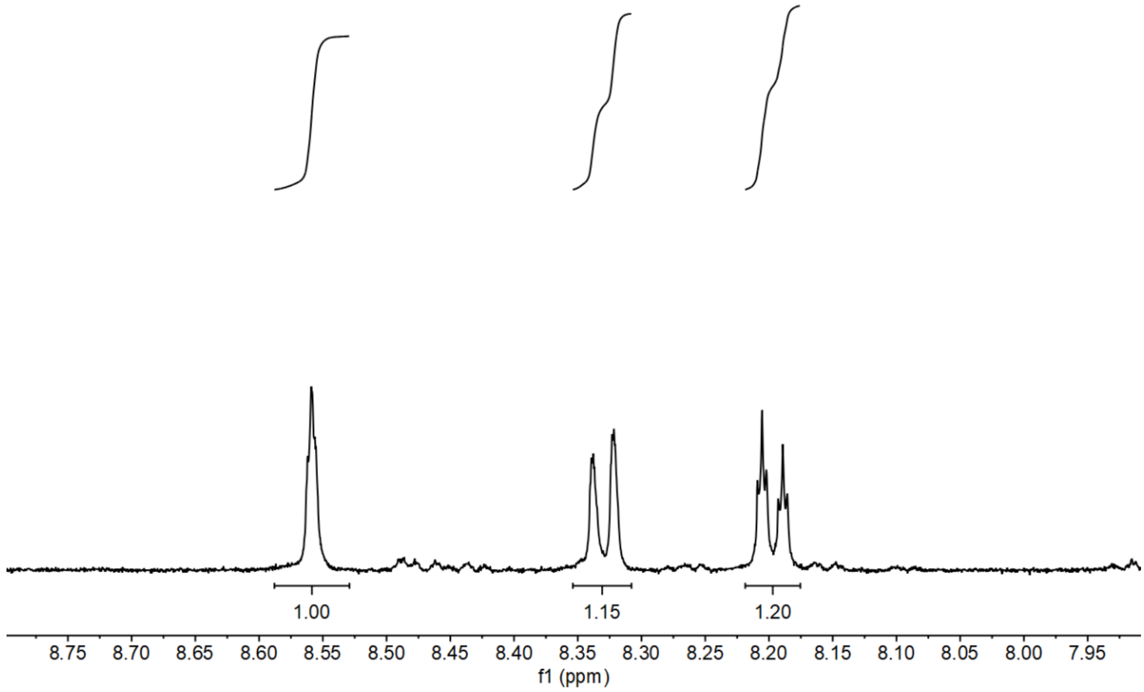

Figure S2- Aromatic region of the ^{1}H NMR spectrum of the purchased AQDS in 10% D_2O.

Characterization Methods:

Nuclear magnetic resonance spectroscopy (NMR):

NMR measurements were performed using a Bruker 500 MHz NMR spectrometer. Samples were prepared in 10% D_2O. 3-(Trimethylsilyl)propionic-2,2,3,3-d_4 acid sodium salt (TSP-d_4) was used as an internal reference.

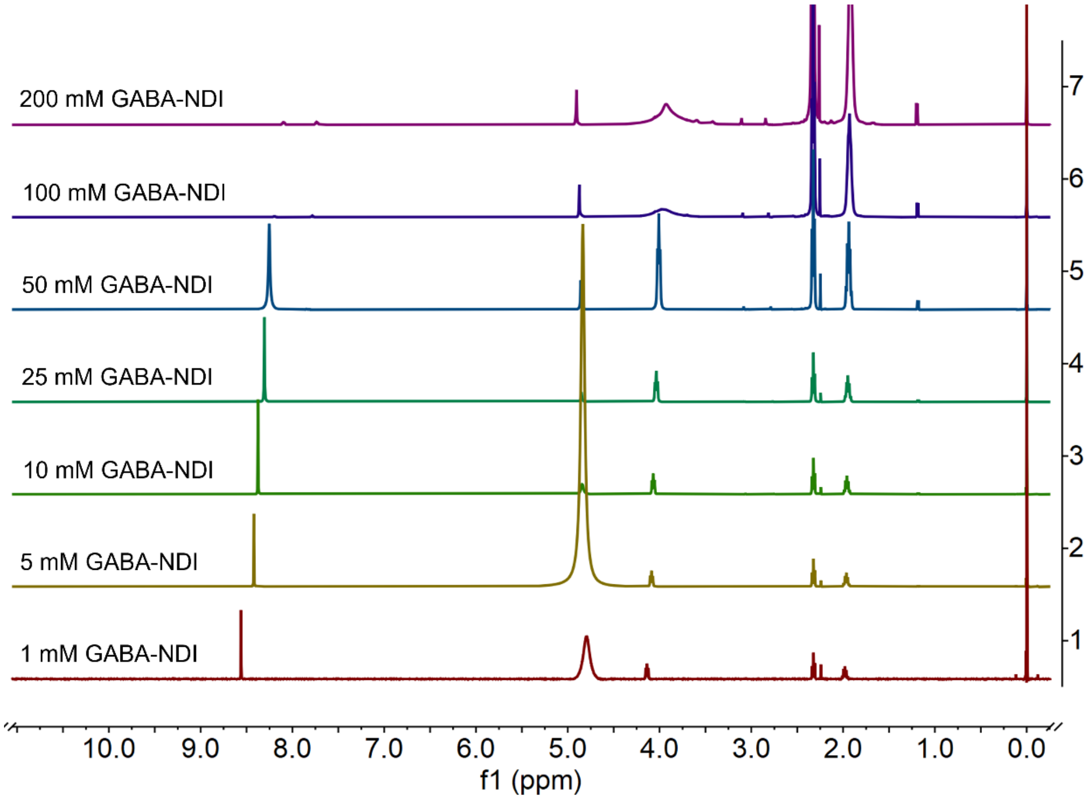

Figure S3- Full ^{1}H-NMR spectra on concentration series of GABA-NDI in 1 M NH_4Cl in 10% D_2O.

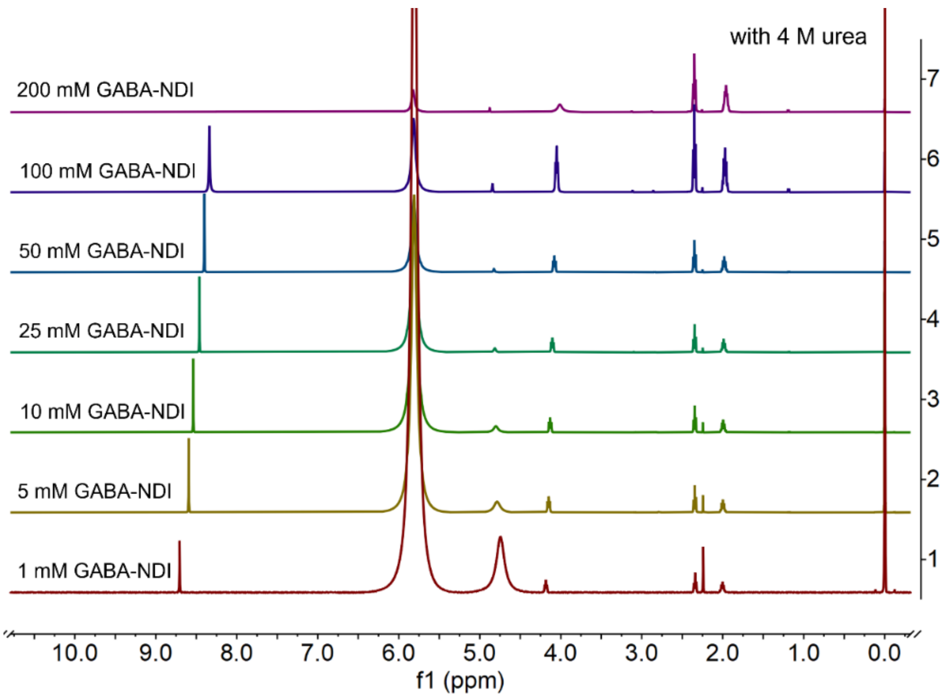

Figure S4- Full ^{1}H-NMR spectra of the aromatic peak for a concentration series of GABA-NDI in 1 M NH_4Cl in 10% D_2O with the addition of 4 M urea.

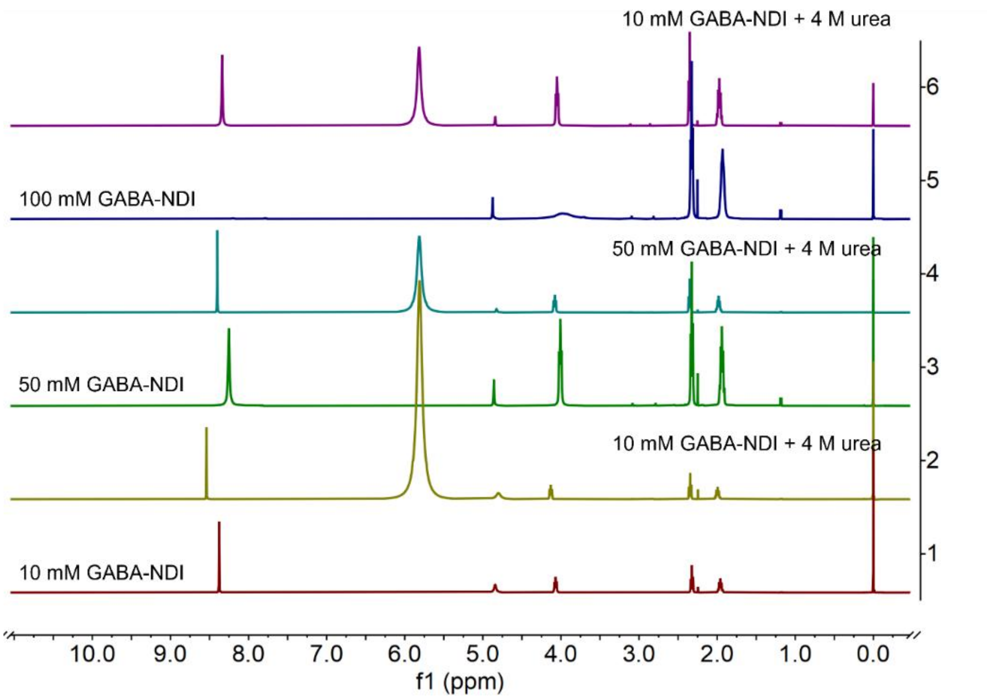

Figure S5- Full ^{1}H-NMR spectra on GABA-NDI (comparison with/out urea addition) in 1 M NH_4Cl in 10% D_2O.

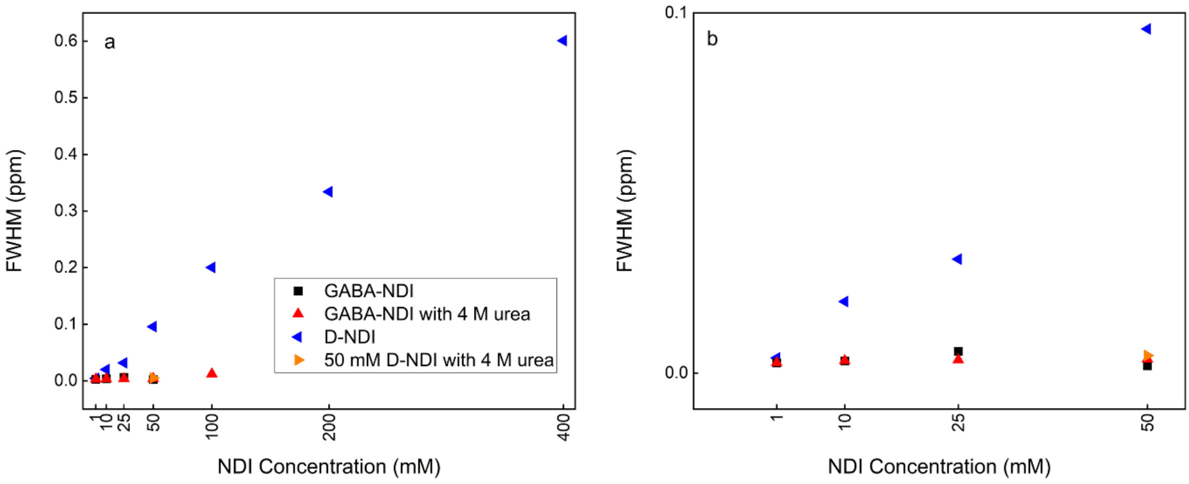

Figure S6- Full width at half maximum (FWHM) of aromatic peak as a function of NDI concentration.

Cyclic voltammetry:

Cyclic voltammetry experiments were conducted using a Gamry Reference 620 potentiostat. A 3 mm glassy carbon working electrode was polished with 0.05 µm alumina slurry and sonicated in DI water for 1 minute prior to each measurement. An Ag/AgCl (3 M KCl) reference electrode from Redox.me (269.3 mV vs. SHE) and a platinum wire counter electrode were used in the three-electrode setup. Cyclic voltammograms (CV) were recorded at the scan rate of 100 mV/s with 90% positive *iR* compensation. All measurements were performed in triplicate and at room temperature.

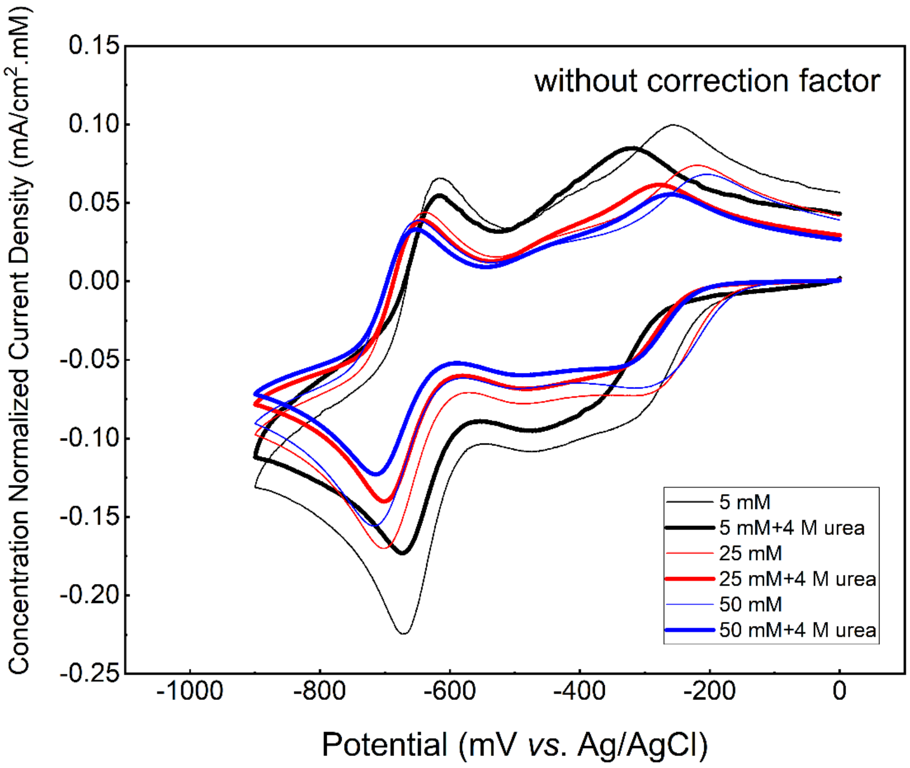

Figure S7- Normalized CV on different concentration of GABA-NDI with 4 M urea in 1 M NH_4Cl at the scan rate of 100 mV/s.

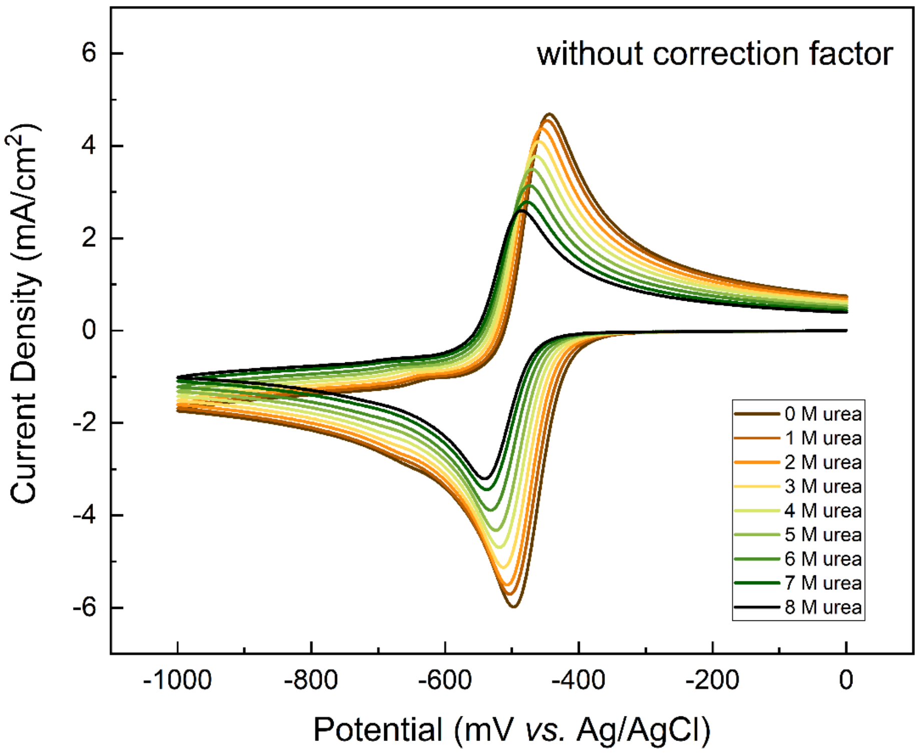

Figure S8- Cyclic voltammograms of 25 mM AQDS in 1 M sodium carbonate buffer pH 9.5 at the scan rate of 100 mV/s.

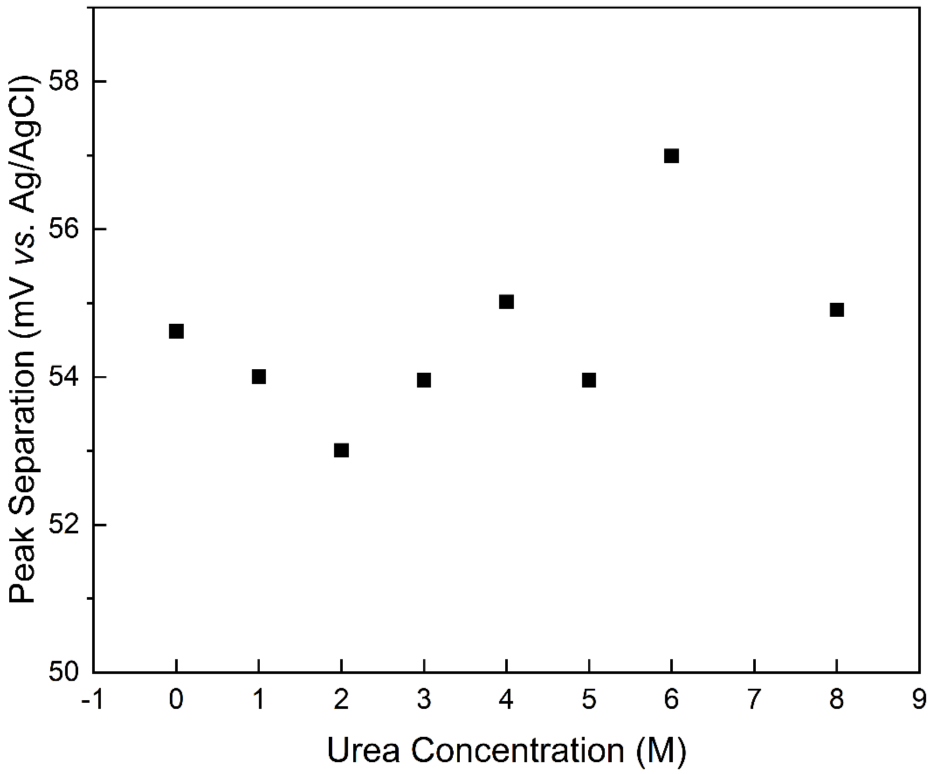

Figure S9- Peak separation of 25 mM AQDS as a function of urea concentration extracted from Figure 8a.

Rotating disk electrode:

Rotating disk electrode measurements (RDE) were conducted using a glassy carbon working electrode (5 mm diameter, Metrohm), which was polished with 0.05 μm alumina slurry (Buehler) and sonicated in deionized water for one minute prior to each measurement. An Ag/AgCl (3 M KCl) reference electrode from Redox.me (269.3 mV vs. SHE) and a platinum wire counter electrode were in the setup. Voltammograms were recorded at a scan rate of 1 mV/s, with rotation speeds of 400, 900, 1600, 2500, and 3600 rpm. A 90% *iR*-compensation was applied using positive feedback. All measurements were performed in triplicate using an Autolab RDE motor in combination with a Gamry Reference 620 potentiostat.

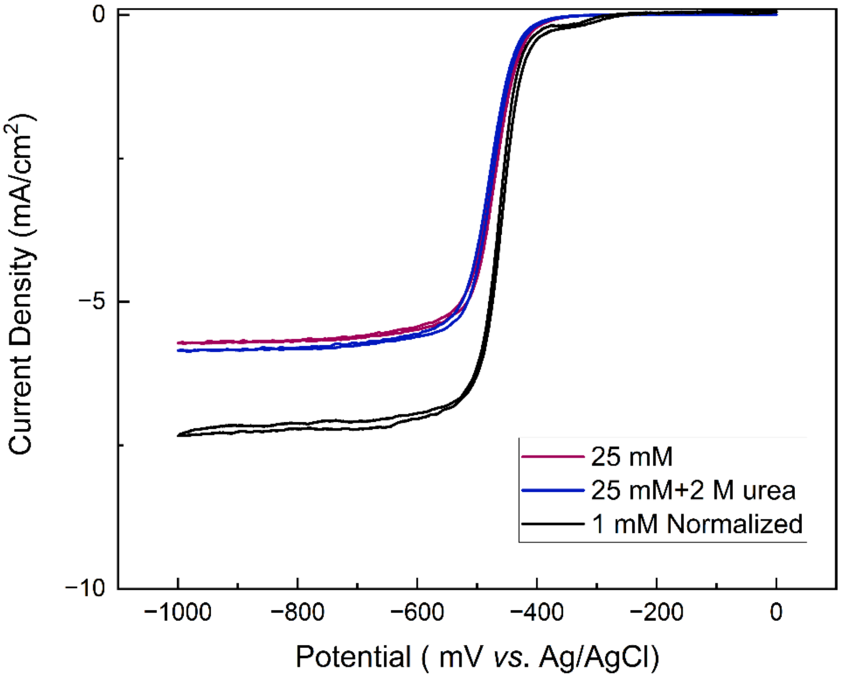

Figure S10- RDE voltammograms of 1 mM (normalized current multiplied by 25) and 25 mM AQDS at 400 rpm and the scan rate of 1 mV/s.

Flow battery measurements:

Electrochemical cycling of the flow batteries was carried out using a lab-built flow cell with flat flow fields and 5 cm^2 heat-treated carbon felt electrodes. The carbon felt, originally 4.6 mm thick, was compressed to 3 mm during cell assembly. PTFE gaskets were used to seal the electrodes. Before assembly, the membrane was pre-soaked in the supporting electrolyte for 2 hours. Electrolyte solutions were pumped through the cell at 30 mL/min using a Chonry BT600M peristaltic pump, calibrated with Masterflex C-Flex tubing (Cole-Parmer). Battery cycling was done using a LANHE 400W battery tester. Electrolytes were prepared with deionized water, and a 2.5:1 volume ratio of positive to negative electrolyte was used to ensure that the negative side limited the overall capacity. The cell was cycled between 1.3 V (charge cut-off) and 0.1 V (discharge cut-off). In addition, the ohmic resistance of the cell at open-circuit potential was measured by electrochemical impedance spectroscopy (EIS) using a BioLogic SP-300 potentiostat, both after one hour of electrolyte circulation (prior to flow battery tests) and after completion of cycling.

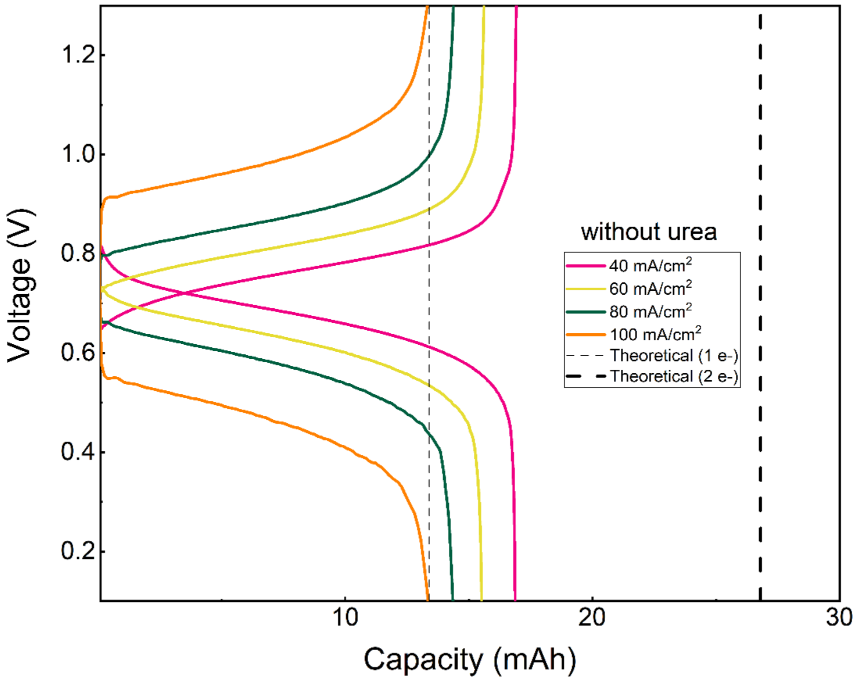

Figure S11- Charge/discharge curves of AQDS/ $Na_4[Fe(CN)_6]$ flow battery (negative side: 20 mL of 25 mM AQDS with 50 mL of 25 mM $Na_4[Fe(CN)_6]$ in 1 M sodium carbonate buffer pH 9.5) without urea.

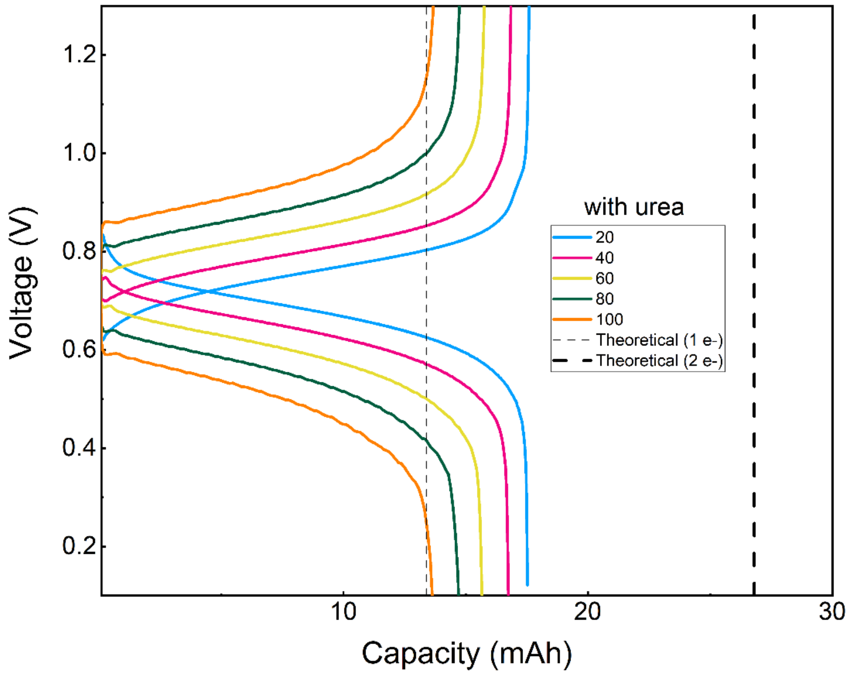

Figure S12- Charge/discharge curves of AQDS/ $Na_4[Fe(CN)_6]$ flow battery (negative side: 20 mL of 25 mM AQDS with 50 mL of 25 mM $Na_4[Fe(CN)_6]$ in 1 M sodium carbonate buffer pH 9.5) with 2 M urea.

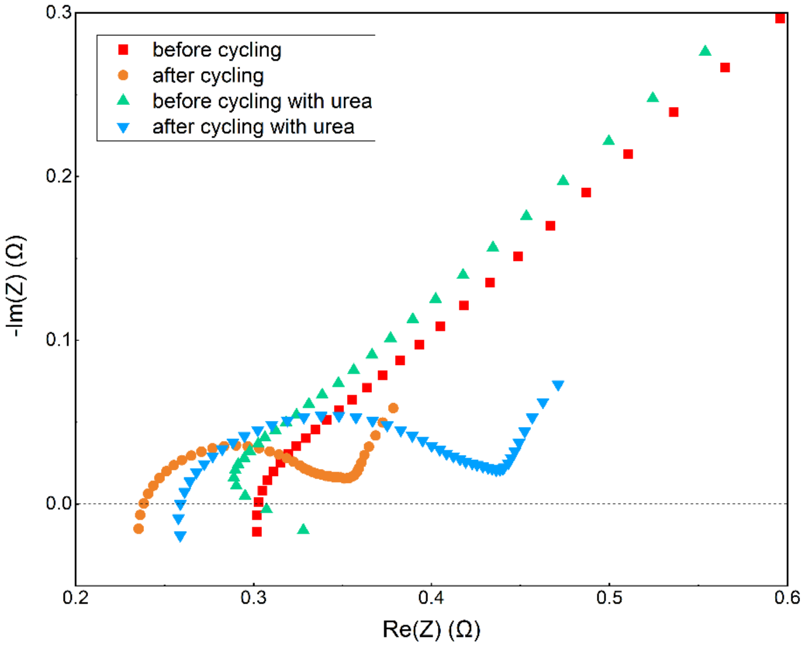

Figure S13- Nyquist plots for EIS of AQDS/ $Na_4[Fe(CN)_6]$ flow battery with and without urea.

Cyclic voltammetry on Ferrocyanide:

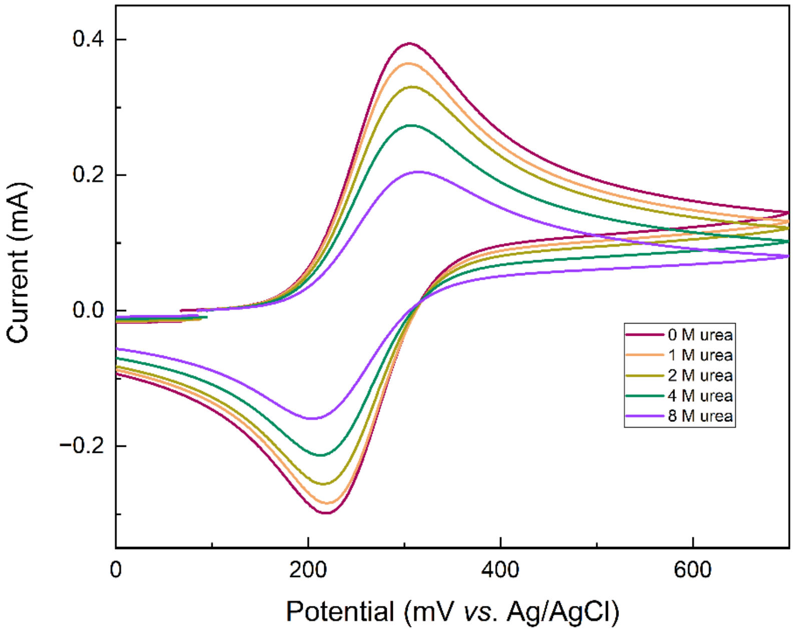

Figure S14- Cyclic voltammograms of 25 mM potassium ferrocyanide in 1 M sodium carbonate buffer pH 9.5 at the scan rate of 100 mV/s.

References

[1] C. Wiberg, F. Owusu, E. Wang and E. Ahlberg, *Energy Technol.*, **2019**, *7*, 1900843.

[2] M. Shahsavan, C. Wiberg and P. Peljo, *Chem. Commun.*, **2022**, *58*, 12692–12695.

[3] T. J. Carney, S. J. Collins, J. S. Moore and F. R. Brushett, *Chem. Mater.*, **2017**, *29*, 4801–4810.
